# Exploring the dynamics of cow milk quality: A bibliometric and scoping review of dairy cattle research

**DOI:** 10.5455/javar.2025.l956

**Published:** 2025-09-22

**Authors:** Randi Mulianda, Santika Anggrahini, Ahmad Sofyan, Zein Ahmad Baihaqi, Novia Qomariyah, Muhammad Ainsyar Harahap, Jhon Firison, Erna Winarti, Hardi Julendra, Hendra Hardian, Andi Ella, Susana Iw Rakhmani, Ririen Indriawaty Altandjung, Harwi Kusnadi, Wulandari Wulandari

**Affiliations:** Research Center for Animal Husbandry, National Research and Innovation Agency (BRIN), Bogor, Indonesia

**Keywords:** Cow, milk quality, bibliometric, Country’s contribution, scopus, sustainability

## Abstract

This study examines the contributions of various countries to milk quality research over the past 27 years (1997–2024) using bibliometric analysis of data retrieved from the Scopus database. The study aims to highlight the efforts required by governments to enhance research in this field. The analysis employs bibliometric tools, including VOSviewer, Scimago Graphica, Bibliometrix, and Microsoft Excel, to visualize data and conduct network analysis. The findings indicate a 13% increase in the number of publications related to milk quality. China leads in the number of publications, with 1,178 documents, whereas Italy excels in journal publications, contributing 188 journals and 388 institutions, accounting for 27.80% of the global total. China also dominates research funding, with 159 institutions representing 26.63% of global funding entities. In terms of citation impact, the United States ranks first with 2,953 citations, followed by Italy, China, and Brazil. This study provides valuable insights into dairy cattle research, particularly milk quality, through bibliometric analysis and a scoping review. The findings can assist stakeholders in identifying research trends, gaps, and innovations, thereby informing strategies to enhance milk production, nutritional quality, and sustainable dairy practices.

## Introduction

The quality of cow’s milk is one of the key factors determining the success of milk production in the dairy industry. Milk quality is not only associated with its nutritional content but also with microbiological factors, animal health, and processing techniques that influence its market value and consumer safety [1,2]. In recent decades, research on cow’s milk quality has advanced rapidly, driven by the need to improve milk production efficiency and ensure the sustainability of the dairy industry. Milk quality has long been assessed through indicators such as somatic cell count (SCC), which remains a key parameter in evaluating udder health and subclinical mastitis. Foundational studies, such as Schukken et al. [3], have established the link between SCC and milk production losses. Moreover, international guidelines, including those outlined by the IDF Bulletin, have set global standards for acceptable milk quality [4].

Several factors that influence cow’s milk quality include feed management, animal health care, barn environment, and disease control, such as mastitis [5,6]. In addition, the composition of milk, including fat content, protein, lactose, and microbiological resistance, also serves as an important indicator in assessing milk quality [7]. Good cow’s milk quality not only ensures a safe product for consumers but also enhances the competitiveness of dairy products in the global market.

Research on the quality of cow’s milk is essential for advancing a sustainable dairy sector [8,9]. Research into milk quality is essential for the advancement of a sustainable dairy sector. High-quality milk is essential for producing premium dairy products and meeting consumer demand [10]. Modern consumers not only expect milk that is safe and nutritious but also products that align with their values regarding environmental sustainability, animal welfare, and ethical production practices [9].

Countries can provide training and skill development initiatives to enhance research quality by fostering collaboration among research institutes, universities, and industry stakeholders. This can be accomplished via research consortia, knowledge exchange initiatives, and forums for information dissemination and experience sharing [11]. Advancements in research on dairy cattle productivity require a multifaceted approach involving the government, industry, and scientific collaboration. Since 1970, milk production per cow in the U.S. has increased significantly, while the number of dairy farms has decreased [12].

However, the declining number of researchers focusing on milk poses a risk to the understanding and management of this vital national resource [13]. Government support is crucial for developing milk production through technical assistance, pasture facilitation, and cattle imports. Additionally, establishing a regulated market and enhancing coordination among stakeholders can boost domestic fresh milk production. The application of omics technology in lactation research has significantly advanced our understanding of physiological processes and may lead to improved nutritional, genetic, and management strategies for dairy cattle [14].

The government needs to prioritize research, as high-quality research is closely linked to national economic growth. Alongside research, education, and training for human resource development also facilitate economic progress [15]. According to [16], high-quality research output contributes to economic growth in both developed and developing countries. This indicates that the government must implement key research policies to ensure a positive impact on the nation.

This study utilizes bibliometric approaches to quantitatively analyze published scientific papers [15]. Bibliometric analysis offers a thorough evaluation of research output [17]. Conducting qualitative analysis presents numerous challenges for researchers, particularly in managing and interpreting complex data [18]. Research on metadata in education and technology can be classified into three primary methodologies: systematic literature review, bibliometric analysis, and meta-analysis [19]. The broad scope of research allows for extensive meta-analysis in the fields of food and agriculture [20–22]. Additionally, a multitude of scholars utilize bibliometric techniques in the realm of meta-analysis.

Despite advancements in cow’s milk quality research, key gaps remain. Most studies focus on nutrition and health, with limited bibliometric and scoping analyses mapping research trends. There is also an imbalance in global research contributions, and few studies explore the impact of emerging technologies or policies on milk quality. This study addresses these gaps through a bibliometric analysis and scoping review to identify trends, research disparities, and future directions.

This study aims to map and analyze the literature related to cow’s milk quality using a bibliometric and scoping review approach. By examining scientific publications published between 1997 and 2024, this research identifies the key factors influencing milk quality and emerging research trends. The selected time span provides a comprehensive overview of the evolution of research in this field, capturing significant advancements in dairy science, technological innovations, and changing quality standards over nearly three decades. Data were obtained from the Scopus database, comprising 1,258 relevant documents, offering an exhaustive summary of the present condition and prospective trajectories of research. This study examines seven distinct research topics to improve comprehension of the subject content.

a) Which country has the highest number of publications?

b) Which country publishes the most journals?

c) Which journal disseminates the most studies regarding the quality of cow’s milk?

d) Which country conducts the most research focusing on cow’s milk quality?

e) Which country has the largest funding institutions?

f) Which countries and authors have the highest citation counts in cow’s milk research?

## Materials and Methods

### Study design

**Figure 1.**
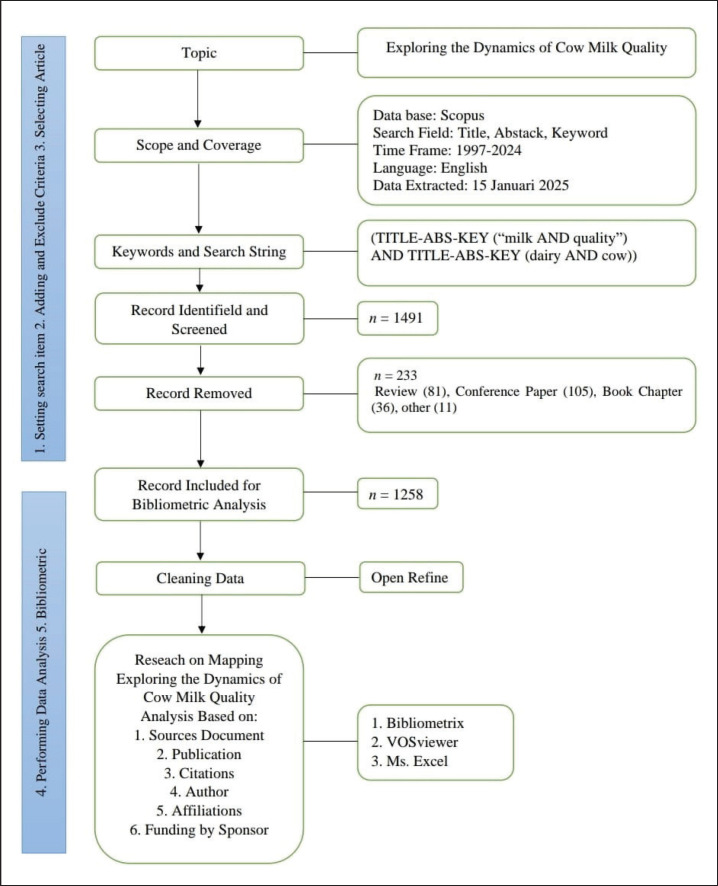
Bibliometric method [24,25].

This study utilizes an iterative methodology to discover pertinent and meaningful keywords, search database sources, and perform precise analysis [23]. This review initiates a comprehensive examination of cow’s milk quality by analyzing contemporary literature, referencing pertinent articles, and monitoring significant characteristics. The study approach incorporates theories from [24,25]. This methodology has six stages: (1) establishing search terms; (2) including and omitting articles; (3) selecting pertinent articles; (4) doing preliminary data analysis; (5) cleansing data with Refine software; and (6) executing bibliometric and network analysis, as depicted in Figure 1. All bibliometric data were retrieved from the Scopus database on January 15, 2025.

The Scopus database serves as a crucial source for bibliometric analysis across various fields, providing broad access to scientific publications. Its comprehensive coverage, dating back to 1966, enables researchers to explore trends, citation patterns, and the evolution of scientific discourse. This overview highlights the key aspects of the utility of the Scopus database in bibliometric studies [26,27]. The specific search terms used to gather data on cow’s milk quality were (“Milk*” AND “Quality”) AND (“Dairy cow”). Using these search terms, we conducted a query in the database and proceeded to the next stage.

### Eligibility criteria

The Scopus database was selected due to its comprehensive indexing of peer-reviewed literature across multiple disciplines, as well as its compatibility with bibliometric tools such as VOSviewer and Bibliometrix. While databases such as Web of Science and PubMed were considered, Scopus provided more consistent metadata formats and broader international journal coverage suitable for this study.

The publication period of 1997–2024 was chosen to capture a comprehensive view of recent trends in milk quality research. The starting point in 1997 reflects the early emergence of modern dairy quality control systems and increasing scholarly output, while including 2024 allows for the most up-to-date analysis. The next process involved selecting articles based on their relevance to the initial search keywords through the application of inclusion and exclusion criteria. To maintain the data’s currency and relevance, only documents published from 1997 to 2024 were included, facilitating the analysis of trends over the past decade. This method adheres to the methodology employed by [28,29]. Figure 1 offers a comprehensive elucidation of the selection criteria utilized. Relevant data, including author names, titles, publication years, affiliations, keywords, funding sources, countries, and citation counts, were compiled in CSV format for subsequent analysis.

From the collected data, we analyzed various statistics, including the yearly distribution of articles and the categorization of publications by year and journal. Assessing the yearly distribution of published papers elucidates patterns in the pertinent literature. Additionally, an analysis of distribution by year and journal was conducted to identify the most active journals publishing research on cow’s milk quality.

The concluding phase of this study methodology entails bibliometric analysis utilizing software such as VOSviewer [30], Bibliometrix [31], Scimago Graphica [32], and MS Excel. This bibliometric analysis aims to identify a country’s contributions by examining various aspects, including authors, affiliations, country of origin, funding institutions, journals, citations, and research collaborations related to the specific topic.

## Results and Discussion

### Analysis of publication

The preliminary statistical analysis demonstrates the yearly distribution of articles, facilitating the assessment of temporal patterns. In general (Table 1), there is a significant upward trend in the number of published articles, particularly in the last decade. The number of publications increased consistently from 2019, peaking in 2024 with 127 articles. In the early years, from 1997 to 2006, the number of published articles remained relatively low, averaging fewer than 20 articles per year. However, since 2010, a more stable increase has been observed, with annual publications ranging between 34 and 57 articles. A significant rise became evident after 2017, reflecting growing attention to the topic of cow’s milk quality. These data indicate a positive development in publication volume, possibly driven by increasing research interest or advancements in dairy science.

Analysis of publication trends over the previous decade, as depicted in Table 1, reveals a substantial rise in the volume of articles concerning cow’s milk quality. Recent studies have highlighted a growing interest in cow’s milk, particularly regarding its impact on health and production [33,34]. The examination of publication data from the Scopus database indicates a consistent annual increase in research articles about cow’s milk. This rise reached its zenith in 2024, culminating in 127 published documents on the subject. The statistics indicate a substantial increase in studies about milk quality, signifying heightened interest and more profound investigation in this domain. Consequently, research on cow’s milk exhibits significant promise for additional exploration. Table 2 indicates an almost 13% rise in studies concerning cow’s milk, underscoring the ongoing expansion and significance of research in this domain.

**Figure 2.**
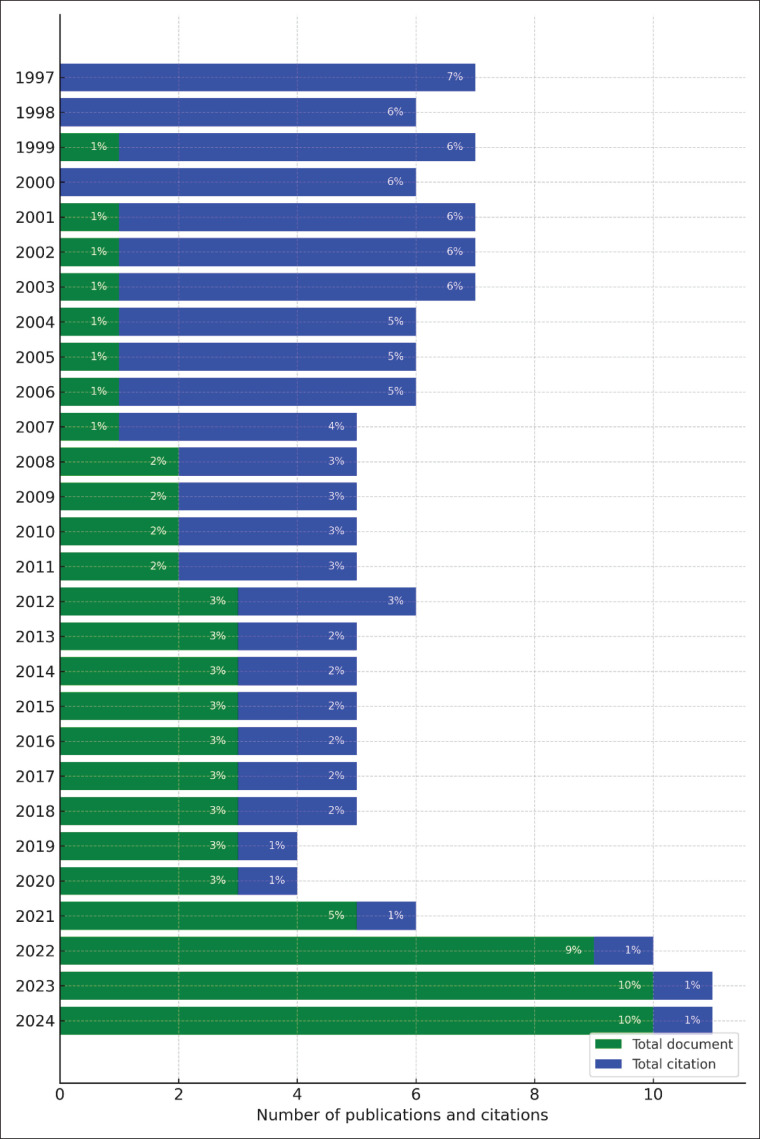
Number of publications and citations about cow milk quality.

Figure 2 depicts the allocation of research about cow’s milk over the preceding decade. The data show a steady growth trend in publications from 1997 to 2024. In the early period (1997–2006), the contribution of publications was relatively small, each accounting for less than 2%, while citation contributions were relatively high, peaking at 7% in 1997. This trend shifted significantly after 2016, when publication contributions began to rise sharply, reaching a peak of 10% in 2024.

**Table 1.** Number of publications.

No	Year	Article	No	Year	Article
1	2024	127	15	2010	34
2	2023	117	16	2009	38
3	2022	98	17	2008	35
4	2021	105	18	2007	25
5	2020	105	19	2006	7
6	2019	93	20	2005	14
7	2018	76	21	2004	15
8	2017	60	22	2003	9
9	2016	57	23	2002	4
10	2015	39	24	2001	10
11	2014	42	25	2000	4
12	2013	47	26	1999	8
13	2012	40	27	1998	6
14	2011	38	28	1997	5

On the other hand, the percentage of citations has shown a gradual decline, particularly in recent years, with near-zero contributions in 2024. This difference is due to the nature of citations, which take time to accumulate, causing newer publications to have fewer citations. This phenomenon suggests that early studies remain foundational and frequently cited [35], while the surge in recent publications reflects a growing interest in cow’s milk research and offers new directions for further exploration in the field.

This trend aligns with the results of investigations undertaken by O’Brien et al. [36], Abrahamsen et al. [37] Cheng [38]. The continuous expansion of research on cow’s milk quality underscores various facets that require further exploration and comprehension, especially among practitioners, academics, and policymakers.

This trend aligns with the findings of studies conducted by O’Brien et al. [36], Abrahamsen et al. [37] Cheng [38]. The ongoing growth of research on cow’s milk quality underscores the many aspects that still require exploration and understanding, particularly among practitioners, researchers, and policymakers.

### Analysis of the number of publications by country

This study particularly highlights the dominant role of China in contributing to scientific publications on cow milk quality, as reflected in Table 2. Following China, other countries with significant contributions include Italy, Brazil, the United States, and the Czech Republic.

**Table 2.** The top 10 percent of documents by country.

No	Country	No. of document	%
1	China	1,178	17%
2	Italy	858	12%
3	Brazil	835	12%
4	USA	469	7%
5	Czech Republic	205	3%
6	France	179	3%
7	India	167	2%
8	Canada	142	2%
9	Sweden	137	2%
10	United Kingdom	137	2%

Research on milk quality has been carried out not only in developed countries but also in developing nations, addressing various aspects of milk production. In developed countries, environmental concerns in dairy farming have attracted increasing attention, especially regarding pollution control and reduction [39,40]. The dairy sector plays a vital global role by providing milk, meat, and organic fertilizer. Generally, developed countries possess better facilities for ensuring adequate livestock nutrition compared to developing countries [41].

In Eastern and Southern Africa, studies have focused on enhancing milk quality and safety, with a particular emphasis on microbiological quality, which is the most frequently examined parameter [42]. Word clouds are used as intuitive visualizations of text data, where the size of each word indicates its frequency. This method has become a popular analytical tool for summarizing and interpreting textual content [43,44]. As shown in Table 2, the ten leading countries in terms of scientific publications on cow milk quality—the top 10 most productive countries in the field of cow’s milk quality—are China, with 1,178 documents, followed by Italy (858), Brazil (835), the USA (469), the Czech Republic (205), France (179), India (167), Canada (142), Sweden, and the United Kingdom (33).

Table 2 indicates a substantial global growth in studies about cow’s milk quality during the previous decade. China accounts for roughly 17% of the total articles produced, with Italy and Brazil each contributing 12%. These data reveals that approximately 15%–20% of cow’s milk research has been impacted by contributions from China, Italy, and Brazil.

China has indeed established itself as a leader in cow’s milk research, driven by significant advancements in various aspects of the dairy industry. The Chinese dairy industry has experienced double-digit annual growth, significantly surpassing the global average of 1% [45]. To enhance cow’s milk quality, countries must prioritize innovation, academic research, and a supportive environment [46]. This multifaceted approach can lead to improved production efficiency, better quality standards, and a stronger global presence in the dairy market.

International collaboration is crucial for advancing dairy cattle genomics and improving feed efficiency globally. Large-scale initiatives such as the Efficient Dairy Genome Project have facilitated data sharing among multiple countries, creating a reference population for joint genomic evaluation [47]. This collaboration is essential for assembling large datasets needed to achieve reliable genomic predictions, particularly for traits with low heritability [48].

### Analysis of the article by sources

**Figure 3.**
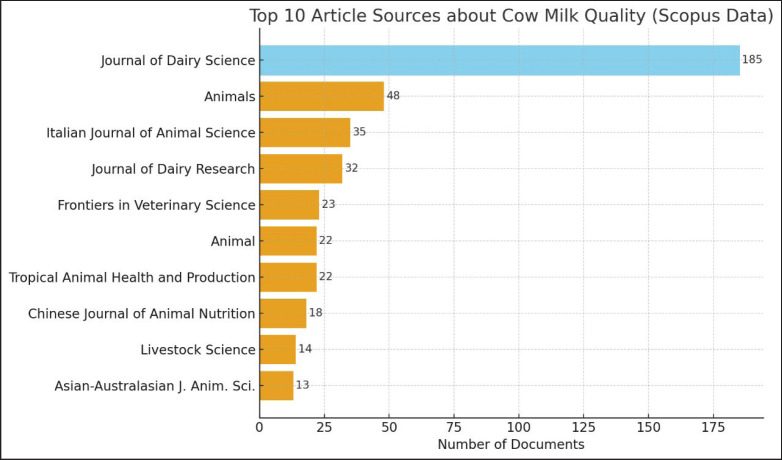
Top 10 article sources about cow milk quality.

Figure 3 displays several scholarly journals relevant to cow’s milk research based on the number of published documents. Five leading journals are highlighted, with the Journal of Dairy Science publishing the most documents (46), making it the primary outlet for studies related to dairy cattle production. It is followed by the Journal of Dairy Research (17 documents), Animal (14), Animals (13), and the Italian Journal of Animal Science (15). These journals serve as key sources that encompass studies on nutrition, genetics, and livestock management, demonstrating their significant contribution to the advancement of cow’s milk research.

In addition to publication volume, these journals also differ notably in their impact factors, which may reflect variations in scope and perceived prestige. For instance, the Journal of Dairy Science (IF = 4.4) and Animal (IF = 4.2) are considered top-tier journals in the field, while the Italian Journal of Animal Science (IF = 2.4) and the Journal of Dairy Research (IF = 1.2) have relatively lower impact factors. These differences suggest that although research productivity is high across multiple journals, the citation influence and international visibility of these publications may vary significantly.

Governments are advised to implement a comprehensive strategy by formulating regulations that emphasize and facilitate research on dairy cow milk quality, foster industrial collaboration, advance educational and training programs, and engage in global knowledge exchange platforms. This strategy will enhance the common progression of knowledge in this vital industry. For instance, the Greener Cattle Initiative [53], a collaborative program, aims to fund research on mitigating enteric methane in dairy cows, addressing environmental issues.

### Affiliate analysis by country

**Figure 4.**
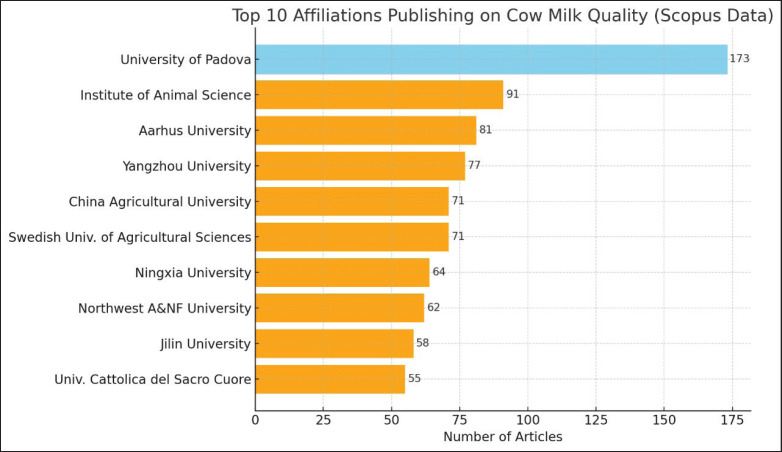
The top 15 affiliations that researched cow milk quality.

Figure 4 illustrates the participation of multiple universities in studies on dairy cow milk quality. The leading 10 institutions are primarily situated in Asia and Europe, especially in Italy and China, reflecting their strong traditions in agriculture and livestock farming. The University of Padova, as a leading contributor, reinforces its role as a global leader in dairy milk quality research. The presence of institutions such as the Institute of Animal Science and Aarhus University further strengthens their position as prominent research centers. Overall, this data demonstrates that dairy cow milk quality research is a global concern, with a primary focus on countries with strong traditions and expertise in milk production.

The University of Padova has established itself as a premier university in dairy cow milk quality, boasting a remarkable total of 173 publications. It is followed by the Institute of Animal Science with 91 articles, Aarhus University with 81 articles, Yangzhou University with 77 articles, and China Agricultural University with 71 articles. Other significant contributors include the Swedish University of Agricultural Sciences with 71 articles, Ningxia University with 64 articles, Northwest A&F University with 62 articles, Jilin University with 58 articles, and Università Cattolica del Sacro Cuore with 55 articles. This data reflects the key role these institutions play in advancing research on dairy cow milk quality, further solidifying their position in the global scientific community.

A competent workforce for research can be created by funding talent development efforts, such as training programs and scholarships. Furthermore, fostering international networking and collaboration, especially with institutions in Italy and China, can enhance the interchange of knowledge and best practices globally. Considering the prominent roles of Italy and China, it is advisable to prioritize the establishment of analogous research institutions by enacting policies that promote and facilitate strategic partnerships with global leaders, emphasizing capacity enhancement and cultivating a culture of research and innovation in dairy cow studies. This aligns with the efforts of genetic consortia in the US, Brazil, and Europe, which are implementing data-driven programs to optimize traits related to production, health, and reproduction [53].

### Country analysis by the author

**Figure 5.**
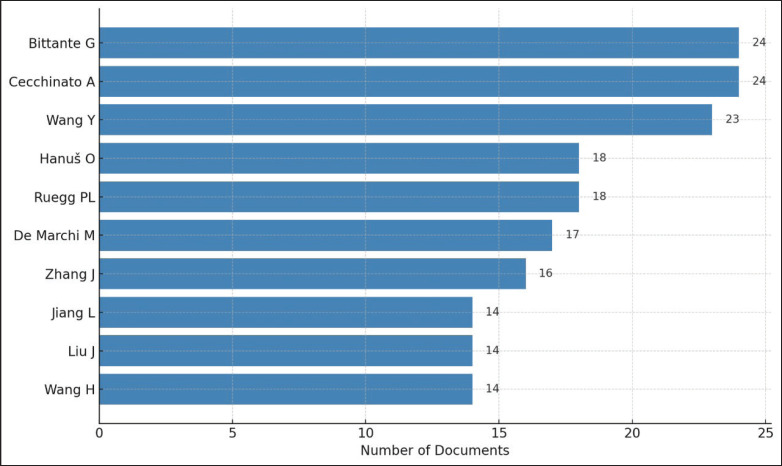
The top 10 authors who researched cow milk quality.

The analysis of research on cow’s milk quality from 1997 to 2024 highlights the significant contributions of authors grouped by their country of origin. This approach enables the identification of countries with high engagement in dairy research based on the productivity of their researchers. The influence of these authors is evaluated by collecting data and recording the number of publications produced or co-authored by each researcher. The top ten authors and their publication counts are presented in Figure 5. Bittante G and Cecchinato A rank highest, contributing 24 articles, followed by Wang Y with 23 articles. Other notable contributors include Hanuš O and Ruegg P.L., each with 18 articles; De Marchi, with 17 articles; Zang J, with 16 articles; and Jiang L, Liu J, and Wang H, each contributing 14 articles.

Italy’s prominence in attracting scholars focused on cow’s milk research offers significant insights for worldwide application. Countries globally can gain from developing and executing talent acquisition and retention strategies designed to recruit specialists in milk quality via research funding, academic roles, and industry collaborations. Italy’s success in dairy research and industry is characterized by a strong production framework, innovative scientific advancements, and a strong emphasis on traditional cheese-making practices. The Italian dairy sector contributes approximately 10% to the agricultural Gross Domestic Product (GDP), with cow’s milk production reaching 10.5 million tons, primarily used for cheese, a hallmark of Italy’s culinary heritage [54].

Establishing international research partnership programs and cooperative projects with Italian professionals can augment knowledge exchange and competence globally. These efforts can be supported through the development of specialized educational programs in cow’s milk quality, the strengthening of partnerships between industry and academia, and investments in modern research facilities focused on this sector. Such measures have the potential to accelerate progress and innovation in the field.

Government assistance is essential for the advancement and viability of the dairy sector. Financial incentives and regulatory frameworks can substantially influence milk production and quality standards [55]. Effective state support can be achieved through various strategies, including industry digitalization and modeling techniques to optimize government regulations [55,56].

Active participation in global networking platforms focused on cow’s milk quality in the livestock industry is essential. Recognizing outstanding contributions can further encourage the formation of a collaborative international research community. Minimizing duplication and enhancing efficiency by connecting scientists across countries is crucial for addressing various challenges in livestock research [57]. The Irish dairy sector serves as a strong example of the benefits of collaboration between research and industry, maintaining high-quality milk production through scientific and technological platforms [58]. Future advancements in the dairy sector will rely on evidence-based information and real-time measures for prediction and decision-making [58].

### Country analysis by funding agencies

The global distribution of funding for cow’s milk quality reflects growing concerns about its safety and nutritional value, influenced by regional practices and consumer expectations. This shift not only emphasizes traditional quality metrics but also broader factors such as environmental sustainability and animal welfare [58]. The globalization of milk consumption, particularly in countries like China, has led to increased scrutiny of milk quality as new markets emerge [59].

An analysis of countries and author affiliations, Table 3, revealed that the majority of contributors to milk quality research are affiliated with academic institutions. Among the top 10 most productive authors identified in this study, nine were affiliated with universities or agricultural colleges, such as the University of Padova (Italy), China Agricultural University, and the University of Wisconsin–Madison. Only one author was affiliated with an industry-based research institution (Dairy Research Institute Ltd., Czech Republic). This finding indicates that the academic sector is the primary driver of research in this area, with relatively limited direct contributions from corporate or industrial entities. It also reflects the ongoing role of public universities and government-supported research centers in advancing dairy science globally.

China’s dominant position in milk quality research, as reflected in publication volume and collaboration networks, can be attributed to several national-level factors. Over the past two decades, the Chinese government has implemented strategic policies to modernize its dairy industry, including substantial investments in dairy science, food safety, and agricultural biotechnology [60]. Programs such as the China Dairy Industry Development Plan 2016–2025, along with strong financial incentives for university-led innovation, have driven increased academic productivity in this field [61]. Furthermore, national funding agencies in China frequently prioritize topics related to food quality and safety, further strengthening scientific output in this area.

 Conversely, the underrepresentation of African countries in this field can be explained by a combination of systemic and structural challenges. Limited national research budgets, inadequate access to high-quality research infrastructure, and reliance on donor-driven agendas may hinder local academic productivity. Moreover, many scientific journals from Africa are not indexed in Scopus, which may contribute to the low visibility of research from the region [62]. This database indexing bias, combined with language barriers and publication fees, poses significant challenges for African researchers in contributing to the global discourse on milk quality [63].

China has made significant contributions to cow’s milk research through various initiatives aimed at improving milk production and understanding milk composition. The dairy sector in Brazil is increasingly focused on enhancing efficiency and sustainability through various innovative practices and technologies [64]. The European Union has significantly influenced cow’s milk research, particularly in improving production efficiency and quality standards [65]. Although the United States ranks fourth, it remains a key player in technological innovation and cattle health research, with a focus on developing milk quality and production efficiency [65,66].

China has demonstrated its leadership as a host to numerous funding institutions supporting cow’s milk research, providing valuable insights for other countries seeking to strengthen their role in this sector. These countries can recognize the importance of establishing dedicated funding institutions that not only encourage innovation but also expand the global knowledge base on the dairy industry. One strategic step that can be taken is to promote public-private partnerships, which can strengthen financial support and ensure that research approaches are more holistic and integrated. Additionally, collaborating with influential countries like China and Brazil, which dominate global funding, offers significant opportunities to combine resources and launch joint initiatives that have the potential to transform the research landscape in this sector.

**Table 3.** Countries and affiliations from the top 10 authors of cow milk quality.

No	Author	Country	Affiliation
1	Bittante, G	Italy	Dipartimento di Agronomia, Alimentazione, Risorse naturali, Animali e Ambiente, University of Padova, PD, Legnaro, Italy
2	Cecchinato, A	Italy	Dipartimento di Agronomia, Alimentazione, Risorse naturali, Animali e Ambiente, University of Padova, PD, Legnaro, Italy
3	Wang, Y	China	College of Animal Science and Technology, China Agricultural University, Beijing 100193, China
4	Hanuš, O	Czech	Dairy Research Institute Ltd., Ke Dvoru 12a, Prague, Czech Republic
5	Ruegg, P.L	USA	Department of Dairy Science, University of Wisconsin-Madison, 1675 Observatory Drive, Madison
6	De Marchi, M	Italy	Department of Agronomy, Food, Natural resources, Animals and Environ-ment, University of Padova, Legnaro 35020, Italy.
7	Zhang, J	China	Key Laboratory of Ruminant Molecular and Cellular Breeding, School of Agriculture, Ningxia University, 750021 Yinchuan, China
8	Jiang, L	China	Beijing Key Laboratory of Dairy Cow Nutrition, College of Animal Science and Technology, Beijing University, China
9	Liu, J	China	College of Veterinary Medicine, Shandong Agricultural University, Tai’an, China
10	Wang, H	China	Beijing Key Laboratory for Dairy Cow Nutrition, Beijing University of Agriculture, Beijing, China

### Analysis of the number of citations

In addition to publication productivity and country-level contributions, citation trends provide valuable insights into the influence and scholarly impact of individual studies. As shown in Table 4, several foundational papers have significantly shaped the field of cow milk quality research. For instance, the article by Pyörälä (2009) in Veterinary Microbiology received 354 citations, while Silanikove (2010) and Keefe (1997) garnered 327 and 284 citations, respectively. These highly cited works predominantly focus on topics such as mastitis, milk microbiology, and somatic cell count—core themes in dairy science. Notably, several top-cited articles originated from North American and European institutions, which may explain the citation leadership of countries like the United States and Canada despite lower overall publication counts compared to China. This reflects the strong influence of early, high-impact studies in shaping ongoing milk quality research.

In addition, Engagement in international research networks, especially with high-citation nations like the U.S. and Italy, offers opportunities for reciprocal learning and joint initiatives that can be very advantageous. Consequently, it is highly advisable for nations to strategically allocate resources towards dairy cow research initiatives, promote international collaboration, endorse publications in prestigious journals, and engage in capacity-building programs. By adopting these strategies, nations can enhance their standing in global research, make significant contributions to the advancement of knowledge in the dairy industry, and foster mutually beneficial partnerships that have the potential to generate more research citations.

**Table 4.** Top 10 number citations by article about cow milk quality research 1997–2024.

No	Article	Total citations	TC per year
1	Pyörälä S, 2009, Vet Microbiol	354	20.8
2	Silanikove N, 2010, Small Ruminant Res	327	20.4
3	Keefe GP, 1997, Can Vet J	284	9.7
4	Schreiner DA, 2003, J Dairy Sci	274	11.9
5	Morand-Fehr P, 2007, Small Ruminant Res	265	13.9
6	Xue M-Y, 2020, Microbiome	247	41.1
7	Makovec JA, 2003, J Dairy Sci	209	9.0
8	Schukken YH, 2009, Vet Microbiol	172	10.1
9	O'callaghan Tf, 2016, J Dairy Sci	170	17
10	Smith Kl, 1997, J Anim Sci	169	5.8

### Analysis of collaboration and network by country

**Figure 6.**
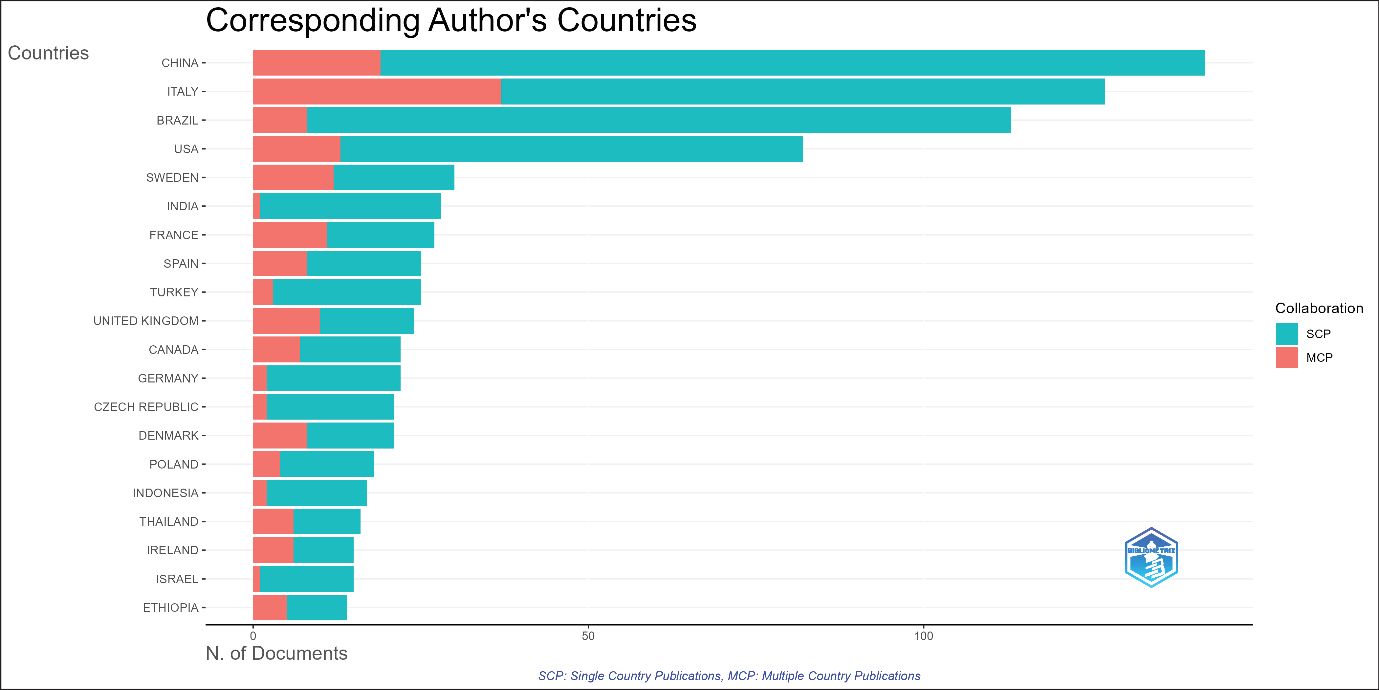
Corresponding author’s countries.

Figure 6 illustrates the publication activity related to dairy cow quality from the perspective of international collaboration over the past decade, Single Country Publications and Multiple Country Publications. China ranks first with a total of 142 articles, 19 of which are the result of collaborations with other countries. Italy ranks second with 127 articles, of which 37 were produced through international partnerships, reflecting a high proportion of collaborative publications. Brazil holds the third position with 113 articles, including 8 articles co-authored with international partners. While China leads in the total number of publications, Italy demonstrates a higher rate of collaboration, emphasizing the importance of global partnerships in enhancing the impact of research on dairy cow quality.

Overall, this figure highlights the significance of cross-border collaboration in expanding knowledge about dairy cow quality. Countries with high research activity can serve as catalysts for promoting global collaboration, which not only strengthens the impact of research but also opens opportunities for underrepresented regions to make more substantial contributions in the future.

In contrast to previous bibliometric reviews that focused on national or regional contexts, such as [37], which examined dairy-related research output in Ireland, this study provides a broader global overview of milk quality research. By analyzing publications across multiple countries from 1997 to 2024, the present review captures long-term trends, international collaboration patterns, and funding dynamics that extend beyond a single geographic focus. Furthermore, this study integrates both bibliometric and scoping approaches, supported by visual tools such as co-authorship maps and keyword clustering, offering a more comprehensive and multidimensional view of the field.

Promoting research exchange programs and enabling institutional alliances are strategic measures to enhance international collaboration [68]. Countries are advised to encourage a collaborative spirit within the academic community by offering sufficient financial backing for international research initiatives and facilitating involvement in academic exchange programs. Furthermore, the development of policies that offer incentives for collaborative research can create a global research ecosystem that supports knowledge exchange, joint initiatives, and innovation in relevant fields. These measures not only strengthen connections between countries but also accelerate scientific progress globally.

The Global Dairy Research Center focuses on advancing knowledge and practices in dairy farming, particularly regarding cattle welfare, nutrition, and milk production efficiency. The research emphasizes the importance of cattle systems, energy requirements, and innovative technologies to improve milking practices. This aligns with the statement by Wu et al. [69], which highlights that optimizing milk production strategies is essential to meet the growing global demand for milk.

### Identifying trends in a word cloud

**Figure 7.**
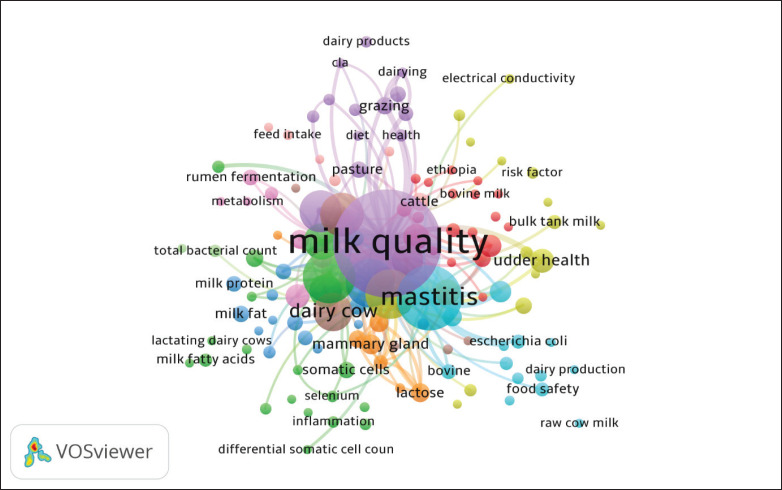
Keyword visualization that often appears in cow milk quality.

Based on Figure 7, a total of 11 clusters are identified. Clusters 1 and 2 each comprise 20 items, while Cluster 3 contains 16 items and Cluster 4 consists of 15 items. Clusters 5 and 6 both include 14 items, whereas Cluster 7 comprises eight items. Additionally, Clusters 8 and 9 each contain seven items, Cluster 10 consists of six items, and Cluster 11 includes two items. Notably, Cluster 1 is centered on milk quality, emphasizing the importance of cleanliness, composition, and the sustainability of dairy production [70–72]. Overall, this visualization reveals the complexity and interconnection of various factors that influence milk quality. Future research could focus on integrating these themes, such as the incorporation of technology and genetics in feed management and livestock health, to produce higher quality, safer, and more sustainable milk.

Cluster 2 consists of keywords such as dairy cattle, dairy cows, differential somatic cells, Holstein cows, inflammation, inflammatory response, lactating dairy cows, melatonin, milk fatty acids, milk production, milk urea nitrogen, nutrition, oxidative stress, parity, performance, polyphenols, selenium, somatic cell score, somatic cells, and vitamin E. This cluster focuses on optimizing rumen fermentation processes to enhance the nutritional content of milk, such as protein and fat levels. This aligns with findings that emphasize the importance of improving rumen fermentation to boost milk quality and nutritional composition [73].

Cluster 3 includes terms such as automatic milking system, breed, Brown Swiss, composition, crossbreeding, fatty acid, genotype, Holstein, metabolomics, milk, milk coagulation, milk fat, milk protein, productivity, quality, and season. The focus of this cluster is on mastitis management to reduce economic losses and improve milk quality [74]. Cluster 4 contains keywords such as body condition score, bovine mastitis, bulk tank milk, clinical mastitis, electrical conductivity, epidemiology, intramammary infection, mastitis pathogens, organic farming, paratuberculosis, risk factors, *Staphylococcus aureus*, somatic cell count, and udder health. Research in this cluster emphasizes the prevention of microbial contamination and risk control in raw milk [75].

Cluster 5 includes terms such as Conjugated Linoleic Acid (CLA), concentrate, dairy products, dairying, diet, forage, grazing, health, heritability, milk composition, milk quality, pasture, silage, and supplementation. This cluster addresses the utilization of technologies, such as machine learning, for milk quality analysis [76,77]. Cluster 6 includes keywords such as animal welfare, bovine, cow’s milk, dairy production, *Escherichia coli*, food safety, machine learning, mastitis, milking, raw cow milk, SCC, *S. aureus*, survey, and treatment. Cluster 6 focuses on metabolomics studies to understand the relationship between inflammation and milk quality [78].

Cluster 7 includes terms such as lactose, lipolysis, mammary gland, milking frequency, protein, urea, cow, and fat. Cluster 8 focuses on the influence of genetics and minerals, such as selenium, on milk quality. Cluster 9 encompasses terms like cows, heat stress, lactation, metabolism, milk productivity, production performance, and rumen fermentation. Cluster 10 includes keywords such as cattle, fatty acid profile, feed intake, fertility, reproduction, and somatic cell counts. Finally, Cluster 11 focuses on the impact of environmental factors and feeding systems on milk quality and composition [79].

Although the term “aflatoxin M1” did not appear in the keyword cluster analysis due to its relatively low frequency in the dataset, it remains a well-known and high-priority topic in food safety research. Its absence in the visualization highlights the need for continued attention to emerging yet critical issues that may not always dominate keyword co-occurrence networks.

As shown in Figure 7, research in dairy cattle and milk production has evolved from a traditional focus on livestock management and animal health to the study of molecular biology, genetics, and the chemical composition of milk. Modern research integrates various omics technologies to enhance production, quality, and animal health in dairy farming [80]. Historical advancements in the dairy industry have led to the development of improved indoor production systems and more effective livestock health management programs [81].

Innovative technologies are increasingly prominent in dairy cattle research, focusing on enhancing production efficiency and developing plant-based alternatives. Advanced automated systems and sensors are utilized to monitor the health of cows, milk quality, and productivity [82]. These technologies enable real-time tracking of various parameters, including feeding habits, rumination, and body temperature. Additionally, research is exploring new methods for producing plant-based milk alternatives using technologies such as ultrasound, high-pressure processing, and pulsed electric fields [83]. These alternatives aim to address issues such as lactose intolerance and environmental concerns.

Sustainability in the dairy industry is crucial for environmental protection and economic viability. Implementing sustainable practices can mitigate the negative environmental impacts through efficient waste management and responsible resource use [84,85]. The industry faces challenges such as stringent regulations, customer expectations, and climate change mitigation measures [85]. However, sustainability indicators are still poorly defined within the industry, making measurement and monitoring a significant challenge [86].

### Research trend analysis and conceptual structure

As shown in Table 5, research in the field of dairy cattle has undergone significant development, particularly in aspects of production, animal health, and milk composition. Based on keyword analysis from various scientific publications, it was found that the main research focus continues to revolve around milk production (milk yield, milk fat) and dairy cattle health (mastitis, somatic cell count), as well as cattle metabolism and diet. The large negative values on the first (Dm1) and second (Dm2) dimensions for keywords such as milk (−0.58, −0.15), dairy cattle (−1.2, −1.2), and mastitis (−1.22, −1.22) indicate that these topics have become foundational in livestock research and have been extensively studied over the past decades. Feedomics, a new field combining various omics technologies, offers new insights into enhancing production, quality, and animal health [87].

However, there is a shift in trends toward the exploration of microbiological, genetic, and experimental aspects in dairy and dairy cattle research. This is evident from the increased relevance of keywords such as *“bacteria” and “micro”* (0.8, 0.8)* and “animal experiment”* (0.85, −1.24), indicating growing attention toward microbiology-based studies and laboratory experiments in evaluating milk quality and animal health. Furthermore, research related to *genetics* (−0.36, −0.36) and *Bos* (0.46, 1.17) suggests that the exploration of dairy cattle genetics is gaining increasing attention, particularly in efforts to enhance productivity and disease resistance [88,89].

The role of somatic cell count in milk quality assessment, as highlighted in earlier works [3], remains relevant in current research trends. The alignment of national dairy practices with global standards, such as those proposed by the IDF Bulletin [4], underscores the importance of harmonizing research outputs with internationally accepted benchmarks.

With the growing awareness of food quality and safety, research on milk microbiology and the biochemical aspects of milk production has been increasingly developing. Recent studies have placed greater emphasis on the role of bacteria in animal health and their impact on milk quality, as reflected by the rising relevance of keywords such as *“microbiology”* (−0.34, −0.34) and *“bacteria” and “micro”* (0.8, 0.8). The WHO 2022 report highlights the importance of microbiological monitoring in the dairy industry to ensure the production of safe and nutritious products for consumers [90].

**Table 5.** Analysis of research trends in the field of cow’s milk (1997-2024).

No	Word	*Dm1	*Dm2	No	Word	*Dm1	*Dm2
1	Milk	−0.58	−0.15	23	Milk.Yield	−1.32	−1.32
2	Female	−0.65	−0.31	24	Bovine.Mastitis	−0.73	−0.73
3	Cattle	−0.82	−0.12	25	Microbiology	−0.34	−0.34
4	Lactation	−0.27	−0.53	26	Milk.Protein	−0.64	−0.64
5	Animals	−0.85	−0.14	27	Veterinary.Medicine	−0.43	−0.43
6	Animal	−0.9	−0.13	28	Mastitis	−1.22	−1.22
7	Article	0.29	−0.72	29	Physiology	−0.06	−0.06
8	Dairying	−0.77	−0.17	30	Udder	−0.86	−0.86
9	Bovine	−0.92	−0.27	31	Somatic.Cell	−1.52	−1.52
10	Nonhuman	0.65	−1.06	32	Food.Quality	−0.57	−0.57
11	Cell.Count	−0.16	−0.93	33	Animal.Food	−0.36	−0.36
12	Dairy.Cattle	0.78	−1.2	34	Lactose	−1.01	−1.01
13	Controlled.Study	0.5	−1.13	35	Mammary.Animal	−0.82	−0.82
14	Chemistry	−0.97	−0.05	36	Cytology	−0.33	−0.33
15	Milk.Production	0.82	−0.94	37	Genetics	−0.36	−0.36
16	Metabolism	−0.95	−0.17	38	Fatty.Acid	−0.15	−0.15
17	Diet	−0.8	−0.1	39	Procedures	−0.15	−0.15
18	Mastitis..Bovine	−0.99	−0.45	40	Cattle.Disease	−0.31	−0.31
19	Animal.Experiment	0.85	−1.24	41	Animal.Feed	−0.17	−0.17
20	Bos	0.46	1.17	42	Bacteria..Micro	0.8	0.8
21	Staphylococcus.A	−0.04	−0.34	43	Milk.Fat	−1.42	−1.42
22	Animalia	0.3	0.88	44	Milk.Proteins	−0.16	−0.16

This shift in trends suggests that research in the field of dairy and dairy cattle is no longer solely focused on traditional aspects such as milk production and livestock diseases but is also beginning to explore new approaches through microbiology, genetics, and laboratory-based experiments. Researchers interested in advancing this field may consider further exploration of milk microbiology and dairy cattle genetics to improve production efficiency and animal health [87].

The first cluster relates to the taxonomy and microbiology of dairy cattle, characterized by keywords such as *Bos*, *Animalia*, and *bacteria/microorganisms.* Research within this cluster primarily focuses on the role of microorganisms in dairy cattle health and milk production. Several studies have shown that microorganisms such as *S. aureus* significantly impact milk quality and can cause mastitis, a disease that adversely affects the livestock industry [91,92]. Furthermore, recent studies emphasize the importance of rumen fermentation in determining the digestive efficiency and metabolism of dairy cattle, which in turn affects milk production and its nutritional content [93].

The increasing focus on technologies such as automated mastitis detection systems reflects a growing alignment between research and industrial needs, particularly in improving animal welfare, milk quality, and early disease diagnosis. This illustrates the importance of technology transfer from academia to industry. Furthermore, the findings of this study are consistent with the goals of SDG 12 Responsible consumption and production by highlighting sustainable research directions that support efficient, safe, and ethical livestock production systems.

The second cluster focuses on dairy cattle health and genetic factors, with keywords such as *mammary glands*, *animal*, *veterinary medicine*, *genetics*, *mastitis, and* *bovine*. Research in this cluster primarily addresses mastitis, one of the most common health disorders in dairy cattle, which has a significant economic impact. Mastitis is often associated with an increased somatic cell count in milk, which reduces milk quality and decreases livestock productivity [94]. Additionally, studies on genetic factors are increasingly developing, particularly in identifying gene variants that can enhance cattle resistance to diseases and improve milk quality [89,95].

The third cluster is related to milk production and quality, indicated by keywords such as *milk production*, *somatic cell*, *dairy cattle*, *milk fat*, and *food quality*. The primary focus of research in this cluster is improving milk production efficiency by considering factors like nutrition, genetics, and livestock health. Several studies have shown that milk quality is heavily influenced by the balance of nutrition and livestock health management, which ultimately affects the fat and protein content in milk [96]. Additionally, with growing awareness of food safety, research into the microbiological quality of milk has become a key area of focus to ensure that milk products are safe for consumption [90].

This conceptual mapping shows that research on dairy cows and milk is evolving beyond just increasing production, expanding into microbiology and biotechnology. Future trends are likely to focus more on the integration of genomic technologies and microbiology to improve cattle health and milk quality. Additionally, research linking gut microbiota health in cows with milk production efficiency is also beginning to develop, highlighting the importance of a holistic approach to animal health and productivity. This shift will likely drive more advanced strategies in livestock management, including personalized nutrition and the use of probiotics or other microbiota-based interventions to optimize both health and milk yield [90,97].

This study did not include retracted publications in the dataset. While the main focus was on trends, collaborations, and productivity in milk quality research, the issue of scientific integrity remains relevant. Retractions—often due to errors, ethical violations, or data manipulation—can affect citation patterns and research credibility. Future bibliometric studies may consider incorporating data from platforms such as Retraction Watch to analyze the frequency, causes, and impact of retracted articles in the dairy science domain.

Keyword analysis revealed that the most frequently occurring terms were directly related to milk composition and quality, including *milk protein*, *milk fat*, *milk yield*, *somatic cell count*, and *food quality*. This indicates that research on milk quality has been a central focus within dairy science over the past two decades. In contrast, keywords associated with broader themes such as *animal welfare*, *greenhouse gas emissions*, or *sustainability* were largely absent from the dataset. This suggests that, while emerging topics are increasingly relevant in dairy research globally, milk quality remains a foundational and consistently explored area in the literature analyzed.

### Bradford’s law analysis

To further strengthen the statistical rigor of this bibliometric study, two classical bibliometric Bradford’s Laws were applied to examine the distribution of scientific output across journals and authors, respectively. Based on Bradford’s Law, a concentration of publications was observed within a limited number of journals. Specifically, a core zone of 10 journals was identified as contributing a significant proportion of the total publications. Among them, the Journal of Dairy Science emerged as the most prolific source, followed by Animals and the Italian Journal of Animal Science. This distribution, visualized in Figure 3, illustrates a steep decline in publication frequency after the core sources, consistent with the typical Bradford curve. The presence of a clearly defined core journal set confirms the field’s reliance on a central cluster of specialized outlets.

Future research in the field of milk quality could benefit from deeper policy-oriented analyses. National dairy regulations such as the European Union’s milk quota system or China’s dairy safety laws have the potential to shape research agendas, funding priorities, and methodological focus across countries. Understanding how these regulatory frameworks influence publication trends may provide important insights into the intersection between science and policy.

In addition, emerging technologies such as lab-grown milk and precision fermentation are rapidly evolving and may significantly disrupt traditional dairy research. These innovations challenge conventional paradigms related to milk composition, safety, sustainability, and consumer acceptance. Therefore, future bibliometric and scoping reviews should consider including alternative dairy technologies and their influence on the direction of milk-related scientific output.

### Limitations

This study is subject to several limitations. First, only English-language articles indexed in the Scopus database were included. While this ensured consistency and relevance for bibliometric tools, it may have led to the exclusion of important non-English publications, potentially limiting the representation of region-specific research. Second, the reliance on Scopus alone may introduce database bias, as it tends to index journals from established publishers and certain regions, which may affect the global generalizability of the findings. Lastly, citation lag is a possible limitation; articles published in recent years (2022–2024) may have lower citation counts due to the short time frame for citation accumulation. Future research may benefit from a multi-database approach, such as combining Scopus with Web of Science or CAB Abstracts, to validate bibliometric trends more comprehensively.

## Conclusion

Bibliometric analysis reveals rapid growth in dairy cow milk quality research over the past two decades, with China leading in publications (1,178 papers) and funding institutions (159 institutions), representing a significant portion of global research efforts. Italy ranks first in journal publications (188 articles) and collaborative research (37 collaborators), establishing a strong presence in the field. The U.S. leads in citations, with 2,953 citations, followed by Italy and China. The findings highlight the importance of global collaboration, government investment, and research infrastructure to address challenges and opportunities in dairy cow milk research.

Future research should focus on enhancing milk quality through smart nutrition, genetic advancements, and milk safety, including the development of probiotics, omega-3 supplementation, and CRISPR technology. Addressing environmental sustainability, such as reducing greenhouse gas emissions and utilizing renewable energy in dairy farming, is also crucial. Additionally, technological innovations such as AI, IoT sensors, and blockchain can optimize dairy operations by improving efficiency, cattle health, and milk quality monitoring. Governments play a key role in supporting research through funding, policy incentives, and regulations to promote higher milk quality standards.

This study’s limitations include its reliance on the Scopus database (1997–2024) and the inclusion of only English-language publications, which may limit the scope of global insights. Future research could benefit from incorporating additional databases (e.g., Web of Science, PubMed) and multilingual studies to provide a broader understanding. Expanding the use of advanced bibliometric tools such as SciMAT, CiteSpace, and Gephi could offer more detailed and sophisticated analysis of research patterns, contributing to the growth of dairy cow milk quality research.

## List of abbreviations

Dm1, dimension 1; Dm2, dimension 2; SCP, single country publications; MCP, multiple country publications; Avg. pub, average publications; TC, total citations.
